# Effect of *equisetum arvense* extract on bone mineral density in Wistar rats via digital radiography

**DOI:** 10.22088/cjim.10.2.176

**Published:** 2019

**Authors:** Nazanin Arbabzadegan, Ali Akbar Moghadamnia, Sohrab Kazemi, Farideh Nozari, Ehsan Moudi, Sina Haghanifar

**Affiliations:** 1 Student Research Committee, Babol University of Medical Science, Babol, Iran; 2 Neuroscience Research Center, Health Research Institute, Babol University of Medical Sciences, Babol, Iran; 3 Cellular and Molecular Biology Research Center, Health Research Institute, Babol University of Medical Science, Babol, Iran; 4 Oral Health Research Center, Health Research Institute, Babol University of Medical Science, Babol, Iran

**Keywords:** *Equisetum arvense*, Osteoporosis, Bone mineral density, Digital radiography.

## Abstract

**Background:**

Osteoporosis is a common disease of old age. The present study used digital radiography to determine the effects of *equisetum arvense* extract on the bone mineral density (BMD) of experimental rats.

**Methods:**

In this experimental study, 25 male and 25 female Wistar rats, aged three weeks old and weighing 100 grams, were randomly divided into five groups: (1) control group, (2) calcium/vitamin D group, (3) 60 mg/kg *equisetum arvense* extract group, (4) 90 mg/kg *equisetum arvense* extract group and (5) 120 mg/kg *equisetum arvense *extract group. Rats received these diets for 30 days. The spongy bone density was measured in the maxilla and mandible using digital radiography and the serum levels of calcium, vitamin D and phosphorus were measured at baseline and after 30 days. The data were analyzed using ANOVA and Tukey test.

**Results:**

There was no significant difference between serum calcium and phosphorus levels in the five groups before and after 30 days. The serum vitamin D in the group receiving calcium and vitamin D was significantly higher than in the other groups (with average values of 24.7, 61.7, 23.47, 23.95 and 39.16 in the male groups 1 to 5 and 29.0, 85.07, 31.58, 42.34 and 18.83 in the female groups 1 to 5, respectively (p<0.001). Moreover, the increased mandibular BMD in the 120 mg/kg *equisetum arvense* group was significantly higher than in the control group (p<0.01).

**Conclusion:**

A diet containing 120 mg/kg *equisetum arvense* extract resulted in increased mandibular bone mineral density.

Achieving peak bone mass is necessary to attain optimal bone health and, hence, reduce the risk of osteoporosis in the future (1). The achievement of peak bone mass will continue until late adolescence or the early 20s (2), depending on the interactions between hormones and growth factors, genetics, physical activity and nutrition, particularly calcium and vitamin D (3-5). Bone mineral density (BMD) can be altered by various factors, such as diet, age and the use of chemical and natural medicines. Osteoporosis is a bone disease characterized by the loss of bone and its structural deterioration. The manifestations of this disease include a reduction in bone mineral content and bone matrix, thus, although the bone is completely normal in terms of the compounds, it is reduced in terms of its content. The reduction of bone mass that occurs in this disease leads to an imbalance between bone loss and its formation and eventually results in demineralization. Osteoporosis causes pain and deformity or bone fractures due to the demineralization of the bone.

Today, much attention is paid to the use of traditional and herbal medicines in the treatment of diseases and, at the same time, researchers attempt to determine the results of using these medicines through standard laboratory techniques. *Equisetum arvense* is a native plant of the Northern Hemisphere that grows in Europe, South Asia, the Himalayas and in countries such as Canada, the United States, Iran, Turkey, India, Korea and Japan (6). 

The use of *equisetum arvense* is a suitable approach to the treatment of osteoporosis because it contains a significant amount of silica and its consumption leads to the absorption and use of calcium and the formation of collagen (4). Due to having factors and compounds such as alkaloids, phytosterols, tannin, triterpenoids and phenolics, *equisetum arvense* is also effective in preventing bone loss that is caused by age and estrogen deficiency (7). In addition to silica, *equisetum arvense* contains secondary metabolites, such as quercetin, kaempferol, luteolin, apigenin, oleanolic acid, betulinic acid and ursolic acid, all of which affect the anabolic activity of osteoblasts (4). In addition, the silica in this plant results in the formation of bone and connective tissue by facilitating the deposition of calcium and other minerals, decreasing the number of osteoclast cells, stimulating the activity of osteoblasts, stimulating collagen synthesis and facilitating the synthesis of glycosaminoglycan and collagen. Despite these effects, few studies have evaluated the results of the effect of *equisetum arvense* in the treatment and prevention of osteoporosis and further research is required in this regard (8). Therefore, it may be possible to use *equisetum arvense* in the treatment of osteoporosis. 

Given the possibility of examining BMD on images through the use of related software and given its lower cost and availability, digital imaging is currently an appropriate option for assessing bone mineral density which has been used in several previous studies (9, 10). 

The main aim of the presented study was to determine the effects of *equisetum arvense *extract on BMD and calcium and phosphorus levels in rats. However, what makes this study different from the research conducted by Kotwal and Badole (2016) is that, this research seeks to investigate the impact of* equisetum arvense *on bone density using radiography, not histopathology methods, as this method of evaluation is cheap, simple, effective, and accurate. In addition to radiographic methods, blood samples are also taken from specimens to evaluate the amount of calcium and phosphorus levels in rats. 

## Methods

This experimental study was approved by the Babol University of Medical Sciences Ethics Committee (code no: MUBABOL.HRI.REC.1396.135). A voucher specimen (0011540) was prepared and is held in the herbarium, Department of Pharmacology, Babol University of Medical Sciences. To prepare the specimens, the upper part of the horsetail plant was separated and then waste parts were removed. The rest of the plant was washed and dried in shade and powdered by an electric mill. The dried horsetail powder was mixed with 70% ethanol and then placed in a shaker for 72 hours. After that, the solution was transferred from filter paper and the hydro alcoholic extract was transferred to the rotary evaporator and the solvent was removed. The present study was carried out to study the effect of extract of *equisetum arvense* in Wistar rats at three levels of dose: L (30 mg/ kg of body weight), M (60 mg/ kg of body weight) and H (120 mg/ kg of body weight) for a period of 30 days, to determine the range of therapeutic dose to be used for the further studies. 

Wistar rats (25 males and 25 females) aged three weeks old and weighing 100 grams were prepared. Each group of 25 male and female rats was randomly divided into five groups of five and each of the five rats was placed is a cage. Both male and female rats have been used for this study to show if there is any significant difference in BMD changes regarding the gender of wistar rats. The study groups included: 

Group 1: Control group with daily diet

Group 2: Daily diet plus 200 mg/kg calcium/vitamin D

Group 3: Daily diet plus 60 mg/kg *equisetum arvense* extract 

Group 4: Daily diet plus 90 mg/kg *equisetum arvense* extract 

Group 5: Daily diet plus 120 mg/kg *equisetum arvense* extract. 

*Equisetum arvense *extract and calcium/vitamin D were given by gavage.

The five groups of rats were then kept at a temperature of 24°C for 30 days under a 12:12 h light-dark cycle in a room with similar conditions and in separate stainless steel cages of a standard size. The first group received the usual daily diet. The second group, as a positive control, received calcium and vitamin D in addition to the normal daily diet. Groups three to five received *equisetum arvense* extract by gavage in addition to the normal daily diet. 

Blood sampling was used to evaluate the effects of *equisetum arvense *on calcium and vitamin D at baseline and after 30 days, while the changes of BMD were investigated using radiography. The following discuss these two different methods in more details.

**Blood sampling: **Blood samples were collected from all of the rats at baseline and at the end of the study. Several 2 cc blood samples were collected from the eyes of the rats using a capillary tube. In the laboratory, the blood samples were placed in a centrifuge device for eight minutes to separate the serum from the blood. The isolated serum was then placed in 2 cc tubes prepared for each animal. For radiography, the rats were first anesthetized with an intraperitoneal injection of 2 ml ketamine (100 mg/ml) and 1 ml of xylazine (20 mg/ml). Blood samples were collected again after 30 days and the calcium, phosphorus and vitamin D levels were measured using an ELISA kit (IDS company for vitamin D and Pars Azmoon for calcium and phosphorus).

**Radiography: **Radiographs of the rats’ heads were then taken using a digital phosphor storage plate (PSP) sensor (occlusal size) in such a way that the lateral image of the jaw could be obtained. The radiographs were taken using a Pro X digital dental imaging device (Planmeca-Helsinki, Finland) under similar conditions in terms of the peak voltage (50 kVp), amperage (8 mA), the distance between the rats and the x-ray tube (50 cm) and the time (0.50 s). 

The PSPs were then processed using PCT (Soredex - Helsinki, Finland) and the spongy bone density was measured at five points for each jaw using Digora for Windows (DfW 2.7). The obtained average value was recorded, as shown in figure 1. In previous studies, this instrument has been frequently used in the evaluation of bone density in small animals such as rats (10-14).

**Figure 1:**
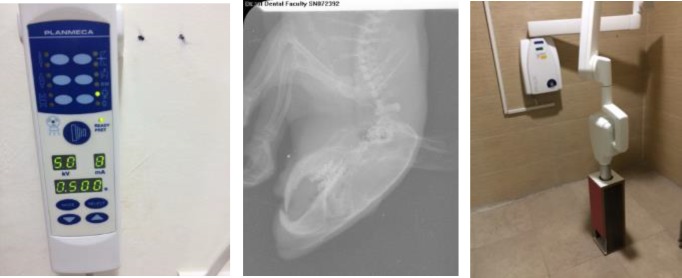
The measurement of rats’ maxillary and mandibular BMD using Radiography

The rats were then anesthetized with ketamine and xylazine and their maxillary and mandibular BMD was measured and recorded using radiography at baseline and after 30 days, as shown in figure 1. Finally, the data were analyzed using SPSS Version 17, the ANOVA test and Tukey’s post-hoc test. 

## Results

The effective compounds of extract were determined using a chemical fingerprint, as shown in table 1. Furthermore, a GC-MS test was done to investigate the content of these active compounds (figure 2). 

Based on the gender of the rats, table 2 shows the mean calcium, phosphorus and vit D levels and the maxillary and mandibular bone mineral densities in the control group, the calcium/vit D group and the groups with different concentrations of *equisetum arvense* extract at baseline and at the end of the study. Tables 3 and 4 present the results of a pairwise comparison between the groups in terms of changes in serum vitamin D and their mandibular BMD before and after consumption of the nutritional diets. The differences in the levels of serum vitamin D before and after 30 days in the control and calcium/vitamin D groups (p<0.001), calcium/vitamin D and 60 mg/kg *equisetum arvense* groups (p<0.001), calcium/vitamin D and 90 mg/kg *equisetum arvense* groups (P<0.001) and calcium/vitamin D and 120 mg/kg *equisetum arvense* groups (p<0.001) were statistically significant (p<0.001). In addition, there was a significant difference in the values ​​of the mandibular BMD in the control and the 120 mg/kg *equisetum arvense* groups (p<0.01), but no significant changes were observed in the other groups.

**Table 1:** The main components of equisetum arvense obtained from a chemical fingerprint

**Peak**	**R.T. min**	**Chemical name**	**% of total**
1	9.696	2-Furancarboxaldehyde	6.093%
2	11.390	5-Hydroxymethylfurfural	27.032%
3	13.295	4-(Ethoxymethyl)phenol	1.365%
4	14.382	ursolic acid	32.918%
5	22.370	1-Oxaspiro[2.5]oct-5-ene	2.508%
6	25.574	oleanolic acid	10.855%
7	26.072	betulinic acid	3.624%
8	27.926	Borane	2.075%
9	33.517	Phosphorus trifluoride	2.222%
10	34.180	Bis(2-ethylhexyl) phthalate	2.772%

**Figure 1:**
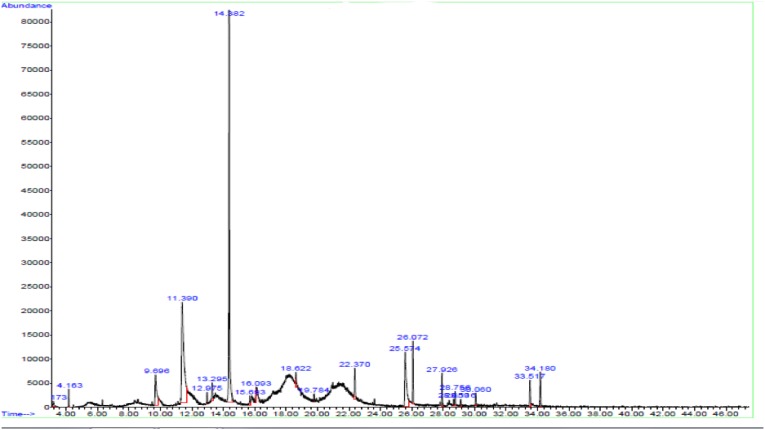
The results of GC-MS test

**Table 2:** Mean levels of BMD and serum calcium, vit D and phosphorus levels before and after 30 days

**After 30 days**					**At baseline**					Group
**Mandibular BMD**	**Maxillary BMD**	**Vitamin D**	**P**	**Calcium**	**Mandibular BMD**	**Maxillary BMD**	**Vitamin D**	**P**	**Ca**	
196.85±2.66	177.5 ± 6.76	24.7 ± 5.88	7.58±0.92	9.03±0.31	189.28 ± 3.3	173.12±5.54	25.85±13.2	9.42±0.52	8.88±0.96	Male, control*
188.0 ± 2.18	168.0±3.81	63.54±18.76	7.04±0.65	8.86±0.29	188.2 ± 1.79	171.52±2.46	28.62±1.82	10.32±0.49	9.76 ±0.33	Female, control*
198.85±2.16	175.25±2.92	61.7 ± 15.85	7.35±0.42	8.93±0.13	185.2 ± 2.87	170.12±2.66	18.4 ± 3.23	10.85±0.66	9.93 ±0.28	Male, Calcium/VitaminD*
195.4 ± 3.59	174.04±2.09	52.07±45.35	6.43±0.35	8.95±0.13	188.1 ± 3.13	174.6 ±6.43	29.75±6.93	9.18±0.94	9.88±0.31	Female, Calcium/VitaminD*
196.44±1.86	170.28±2.46	23.47 ± 6.49	8.6 ± 1.66	9.47±0.25	184.64 ± 3.04	167.56±1.69	24.9±12.39	10.03±0.05	10.35±0.3	Male, 60 mg/kg Equisetum Arvense
193.44±3.31	171.36±4.34	31.58 ± 8.51	6.78±0.54	8.56±0.52	187.68 ± 2.0	168.88±1.22	23.04±1.99	9.62±0.43	10.14±0.75	Female, 60 mg/kg Equisetum Arvense
198.7 ± 5.18	176.5 ± 4.87	23.95 ± 8.43	7.88±0.22	8.73±0.43	182.84 ± 2.64	169.88±1.72	18.72±1.92	9.62±0.97	10.06±0.55	Male, 90 mg/kg Equisetum Arvense
195.0 ± 4.07	174.3 ± 1.55	42.34 ± 4.23	6.32±0.65	9.18±0.28	190.32 ± 2.99	173.56±2.74	28.16±6.87	10.22±0.84	9.22±0.31	Female, 90 mg/kg Equisetum Arvense
193.36±3.21	169.6 ± 1.91	39.16 ± 4.98	6.78±0.73	8.42±0.56	177.56 ± 4.32	161.4±6.62	32.02 ± 4.1	9.78±0.76	9.18±0.28	Male, 120 mg/kg Equisetum Arvense
197.4±13.03	179.0±17.03	18.83 ± 0.29	8.77±0.55	8.93±0.25	178.52 ± 3.64	163.52±3.95	28.32±7.69	9.97±1.11	9.2 ±0.51	Female, 120 mg/kg Equisetum Arvense

**Figure 3:**
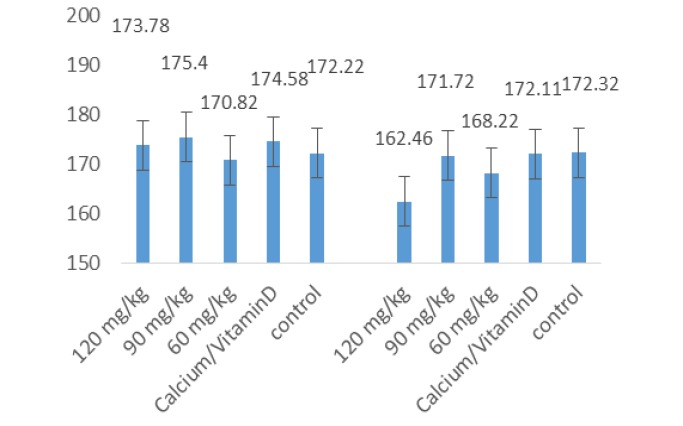
The mean levels of mandibular BMD at baseline and after 30 days

**Figure 4:**
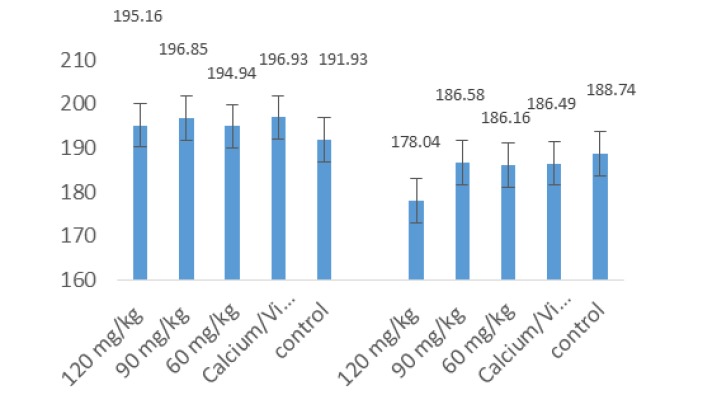
The mean levels of maxillary BMD at baseline and after 30 days

The mean levels maxillary and mandibular BMD at baseline and after 30 days are shown in figures 3 and 4. According to the results of the Tukey test, the mean difference in serum vitamin D in the calcium/vitamin D group was significantly higher than in the control group and the groups receiving *equisetum arvense* extract (p<0.05). The maxillary BMD decreased in animals receiving 60 and 90 mg/kg *equisetum arvense* in comparison with the control group and the group receiving calcium/vitamin D but these reductions were not significant and an increase in 120 mg/kg *equisetum arvense* group was not significant.

* These groups are shared with a research project (code no: 964505) conducted at the Babol University of Medical Sciences.

With regard to the mandibular BMD, only the density in the 120 mg/kg *equisetum arvense* group was significantly higher than the control group (p<0.05) but the BMD changes were not significant in the other groups. With regard to the mandibular BMD based on gender, the density in males and females and in the animals in the 120 mg/kg *equisetum arvense* group increased compared to the control group and the group receiving calcium/vitamin D and this increase was only significant in comparison with the female control group (p<0.05).

**Table 3:** Mean and standard error of changes in vitamin D levels

**P-value**	**Standard error**	**Mean changes**	**Second group**	**First group**
0.001	5.35	21.51	Calcium/Vitamin D	Control
NS	5.16	12.78	60 mg/kg *Equisetum Arvense*	Control
NS*	5.0	11.19	90 mg/kg *Equisetum Arvense*	Control
NS	5.16	12.83	120 mg/kg *Equisetum Arvense*	Control
0.001	5.49	34.29	60 mg/kg *Equisetum Arvense*	Calcium/Vitamin D
0.001	5.35	32.7	90 mg/kg *Equisetum Arvense*	Calcium/Vitamin D
0.001	5.49	34.34	120 mg/kg *Equisetum Arvense*	Calcium/Vitamin D
NS	5.16	1.59	90 mg/kg *Equisetum Arvense*	60 mg/kg Equisetum Arvense
NS	5.31	0.05	120 mg/kg *Equisetum Arvense*	60 mg/kg Equisetum Arvense
NS	5.16	1.64	120 mg/kg *Equisetum Arvense*	90 mg/kg Equisetum Arvense

* = Not significant

**Table 4:** Mean and standard error of changes in the mandibular BMD of rats

**P-value**	**Standard error**	**Mean changes**	**Second group**	**First group**
NS*	3.59	6.37	Calcium/Vitamin D	Control
NS	3.4	4.45	60 mg/kg *Equisetum Arvense*	Control
NS	3.59	5.22	90 mg/kg *Equisetum Arvense*	Control
0.01	3.48	13.16	120 mg/kg *Equisetum Arvense*	Control
NS	3.5	1.92	60 mg/kg *Equisetum Arvense*	Calcium/Vitamin D
NS	3.69	1.15	90 mg/kg *Equisetum Arvense*	Calcium/Vitamin D
NS	3.59	6.79	120 mg/kg* Equisetum Arvense*	Calcium/Vitamin D
NS	3.5	0.77	90 mg/kg *Equisetum Arvense*	60 mg/kg Equisetum Arvense
NS	3.4	8.71	120 mg/kg *Equisetum Arvense*	60 mg/kg Equisetum Arvense
NS	3.59	7.94	120 mg/kg *Equisetum Arvense*	90 mg/kg Equisetum Arvense

* = Not significant

## Discussion

According to the research conducted by previous authors, ursolic acid (32.918%) and oleanolic acid (10.855%) extracted in equisetum arvense can improve bone properties, calcium balance, and modulate vitamin D metabolism. Ursolic acid and oleanolic acid have been reported to stimulate osteoblastic differentiation and inhibit osteoclast formation in vitro (15, 16). 

The mandibular BMD in the 120 mg/kg *equisetum arvense* group was significantly higher than in the control group but there were no significant differences when compared with the other groups. In addition, the BMD increased in both male and female subjects and in the 120 mg/kg *equisetum arvense* group compared to the control group and the group receiving calcium/vit D. This increase was only significant compared to the control group of female animals. There was no significant difference in the serum calcium and phosphorus levels and the mean difference at the baseline and after 30 days. However, the mean difference in serum vitamin D in the group receiving calcium/vit D was significantly higher than the control group and the groups receiving *equisetum arvense* extract. 

The decrease in the serum calcium levels is due to increased calcium excretion and reduced absorption in the body. Silica can increase bone mineralization by reducing the rate of calcium excretion. However, the reduction in the serum calcium levels of the animals receiving a normal diet along with *equisetum arvense* extract was not significant and acceptable in the present study. In the study of Kotwal and Badole (2016), the use of *equisetum arvense* extract did not significantly change the serum calcium levels, which is consistent with the results of the present study (3). In this study, the highest serum vit D levels were observed in animals receiving a calcium/vit D diet and the increase in the serum vit D levels in animals receiving calcium/vit D was significantly higher in the control group and the 60, 90 and 120 mg/kg *equisetum arvense *groups. Therefore, receiving different concentrations of *equisetum arvense* extract has a lower effect on increasing the serum vit D levels compared to the usual calcium/vit D diet. However, it is necessary to evaluate other concentrations of the plant in this field and to report the results.

In the study conducted by Kotwal and Badole (2016), the increase in the thickness of the cortical and cancellous bones in the group receiving *equisetum arvense* extract (which was also evident in histological observations), indicates the bone mineralization ability and activity of the plant (3). Meanwhile, the use of calcium and vit D supplements, along with *equisetum arvense*, decreased osteoporosis. In the present study, the consumption of *equisetum arvense* extract, especially at a concentration of 120 mg/kg, increased the mandibular and maxillary BMD. On the other hand, an investigation by Menghini et al. (2016) of the effects of using a natural formulation containing lactoferrin, equisetum arvense soy isoflavone and vit D3 in bone remodeling and inflammatory markers in rats demonstrated that this formulation was useful in preventing and treating osteoporosis (2). 

Furthermore, Corletto (1999) investigated the absorption rate of silicone from an *equisetum arvense* diet in postmenopausal women who suffered from osteoporosis. The results of the study showed that treatment with *equisetum arvense* extract and calcium supplements were effective in improving bone metabolism (8). These observations were also reported in the present study on *equisetum arvense* extract, especially at a concentration of 120 mg/kg. 

In conclusion, the objective of the present work was to study the effect of extract of *equisetum arvense* in wistar rats at three levels of dose: L (30 mg/ kg of body weight), M (60 mg/ kg of body weight) and H (120 mg/ kg of body weight) for a period of 30 days, to determine the range of therapeutic dose to be used for the further studies.

This study found that 120 mg/kg *equisetum arvense* extract resulted in a significant increase in mandibular BMD but the BMD changes were not significant in the other groups. With regard to the mandibular BMD based on gender, the density in males and females and in the animals in the 120 mg/kg *equisetum arvense* group increased compared to the control group and the group receiving calcium/vitamin D and this increase was only significant in comparison with the female control group. . However, there is still a need for further studies on *equisetum arvense* extract at other concentrations.
